# Usage of Accessory Anteromedial Portal for Anatomical Placement of Femoral Tunnel in Transportal Anterior Cruciate Ligament Reconstruction: A Prospective Study

**DOI:** 10.7759/cureus.17095

**Published:** 2021-08-11

**Authors:** Harshal Sakale, Alok C Agrawal, Mukund Madhav Ojha, Bikram Kar, Rakshit J, Ranjeet Choudhary

**Affiliations:** 1 Department of Orthopaedics, All India Institute of Medical Sciences, Raipur, Raipur, IND

**Keywords:** anterior cruciate ligament, accessory anteromedial portal, transportal, arthroscopy, anatomical reconstruction

## Abstract

Background and objective

The anterior cruciate ligament (ACL) has an essential role in preserving the function and stability of the knee joint. It acts primarily to prevent anterior tibial translation. Arthroscopic ACL reconstruction using quadrupled hamstring graft is the widely practiced modality for treating ACL injuries nowadays. The objective of this study was to assess the functional outcomes of ACL reconstruction using the transportal approach for a femoral tunnel through an accessory anteromedial portal (AAM).

Materials and methods

This prospective study included 35 patients who met the inclusion criteria. All patients underwent arthroscopic reconstruction of ACL using quadrupled hamstring tendon graft via transportal technique for femoral canal reaming through AAM. Patients were assessed for the functional outcome for a year using the Tegner-Lysholm knee scoring system.

Results

The analysis of the studied cases revealed that the mean age of the patients was around 27 years. Males were affected more than females. The left side (77.14%) was affected more than the right side (22.86%); 54.28% of patients had a history of road traffic accidents (RTAs). Preoperatively, 19 (54.28%) patients had poor and 16 (45.72%) patients had fair Tegner-Lysholm scores. After one year of follow-up, 29 (82.85%) of the patients had an excellent score as per the Tegner-Lysholm scoring system. Three patients had knee pain and thigh muscle wasting (2-3 cm), and two of them also had a sense of giving away during follow-up.

Conclusion

Anatomical reattachment of tendon graft for ACL reconstruction at femoral and tibial footprints is indispensable for good functional outcome and knee kinematics, and the usage of the AAM provides good visualization of femoral footprint and ease to surgeons during ACL reconstruction for making a near-accurate femoral tunnel and thereby achieving better outcomes.

## Introduction

The anterior cruciate ligament (ACL) is the most commonly damaged structure of the knee after a traumatic injury [1]. Along with all other ligament structures, the ACL plays a vital role in preserving the function and stability of the knee joint. It acts primarily to prevent anterior tibial translation. The ACL-deficient knee has altered knee kinematics, which predisposes joints to future degenerative changes [2,3]. With 100,000 of these knees being repaired yearly, the annual occurrence of ACL insufficiency is over 200,000 cases. Approximate 70% of ACL trauma is caused by non-contact mechanisms, while the remaining 30% occurs from direct contact. ACL trauma should be intensively studied because of its high prevalence, and the outcomes of ACL surgery attract huge interest [4,5].

Arthroscopic ACL repair is the widely accepted modality for the treatment of ACL injuries nowadays. Traditionally, extra and intraarticular reconstructions were conducted by open arthrotomy, but most surgeons favor arthroscopic anterior cruciate ligament reconstruction with the modern understanding of biomechanics and with the current availability of advanced equipment and implants [6]. The benefits of the arthroscopic approach include minimal skin and capsular incisions, enhanced viewing of the intercondylar notch for tunnel making, less postoperative discomfort, fewer adhesions, and earlier mobility and rehabilitation [7]. Drilling a femoral tunnel at the anatomical location is essential for a good outcome. Various studies in the literature have described two methods for tunnel placement: transportal and transtibial. ACL reconstruction using the transportal technique helps in better anatomical placement of the femoral tunnel, but there are certain disadvantages such as posterior wall breakout, short femoral tunnel, and graft breakage due to stress while using transportal technique [8]. Multiple studies have endorsed the effectiveness of anatomical ACL reconstruction in preserving normal biomechanics of the knee [9]. In our institute, we follow the transportal approach for arthroscopic ACL reconstruction, which is presently advocated by most of the studies. The objective of this study was to assess the functional outcomes of ACL reconstruction using the transportal approach for a femoral tunnel through an accessory anteromedial portal (AAM) and also to determine the importance of making an AAM.

## Materials and methods

Study setting

The present study was conducted in the Department of Orthopaedics at a tertiary care center. It was a prospective hospital-based study that enrolled a total of 38 cases with symptomatic ACL insufficiency as a result of trauma. Patients in the age group of 18-45 years, with clinical and MRI evidence of ACL tear, were included. Patients with grade 1 ACL injuries and those who had associated fractures and multi-ligament involvement, any prior surgery, and those with signs of infection, and those who were lost to follow-up were excluded from the study.

Methods

**Figure 1:**
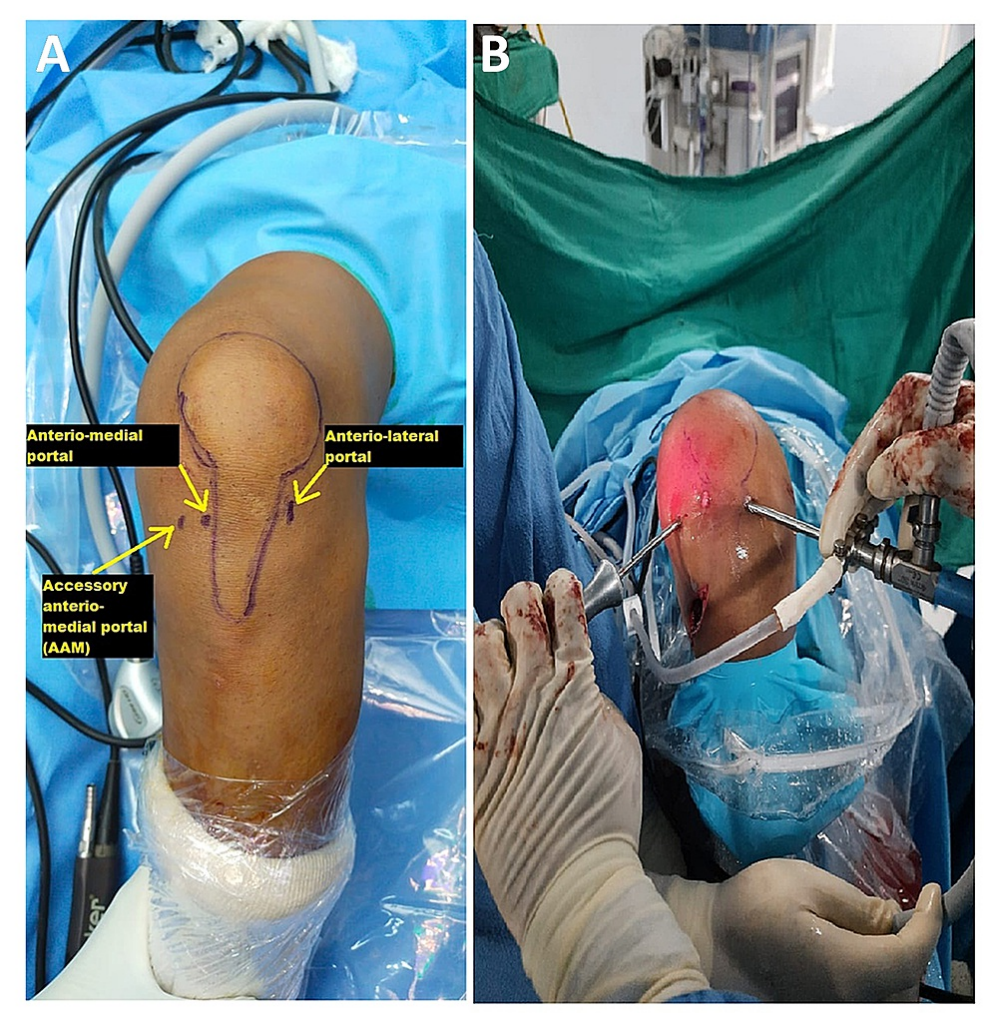
(A) Standard knee arthroscopy portals and position of AAM. (B) Instrumentation through AAM during ACL reconstruction AAM: anteriomedial portal; ACL: anterior cruciate ligament

**Figure 2:**
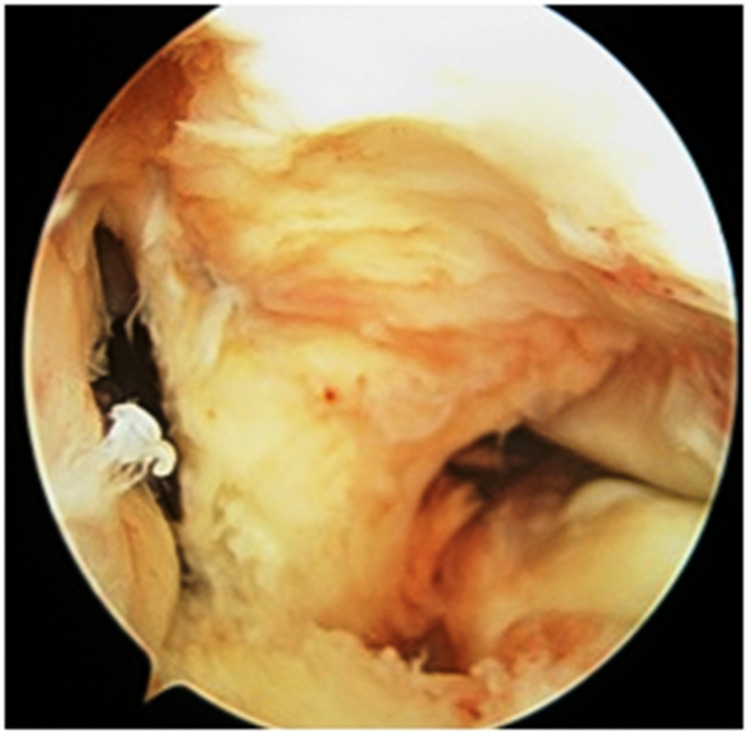
Diagnostic arthroscopy (complete ACL tear) ACL: anterior cruciate ligament

Patients with clinically and MRI-proven ACL insufficiency were admitted to the orthopaedics ward and prepped up for surgery. All patients were operated on under spinal-epidural anaesthesia. Under anaesthesia, the anterior drawer test, posterior drawer test, Lachman test, and pivot shift test were performed. Under tourniquet control, the arthroscopic reconstruction procedure was performed. Standard arthroscopic portals and AAMs were established. AAM is established using an 18G spinal needle and simultaneous visualization under the arthroscope, which should be placed as low as possible so that the instrumentation does not injure the medial femoral condyle; the position usually comes approximately 1.5-2 cm medial and 1.5-2 cm below the standard anteromedial portal. Figure 1A shows the placement of standard arthroscopic portals and AAM, and Figure 1b shows instrumentation through AAM during ACL reconstruction. Diagnostic arthroscopy was performed to evaluate the entire knee joint for any associated injuries (Figure 2).

**Figure 3:**
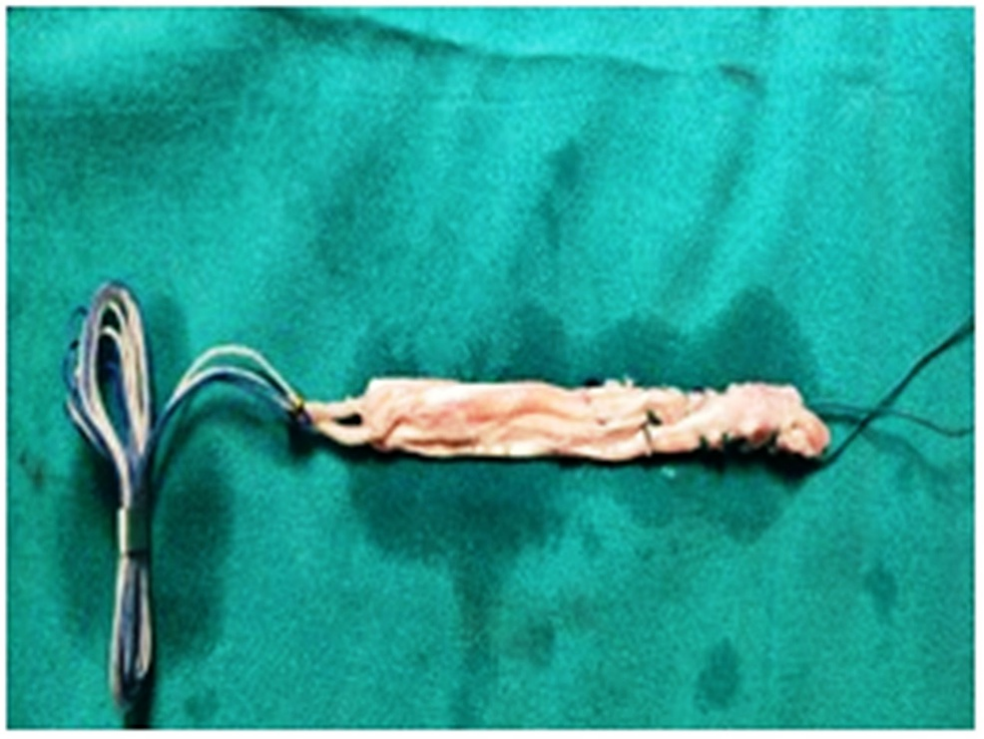
Quadruple graft

**Figure 4:**
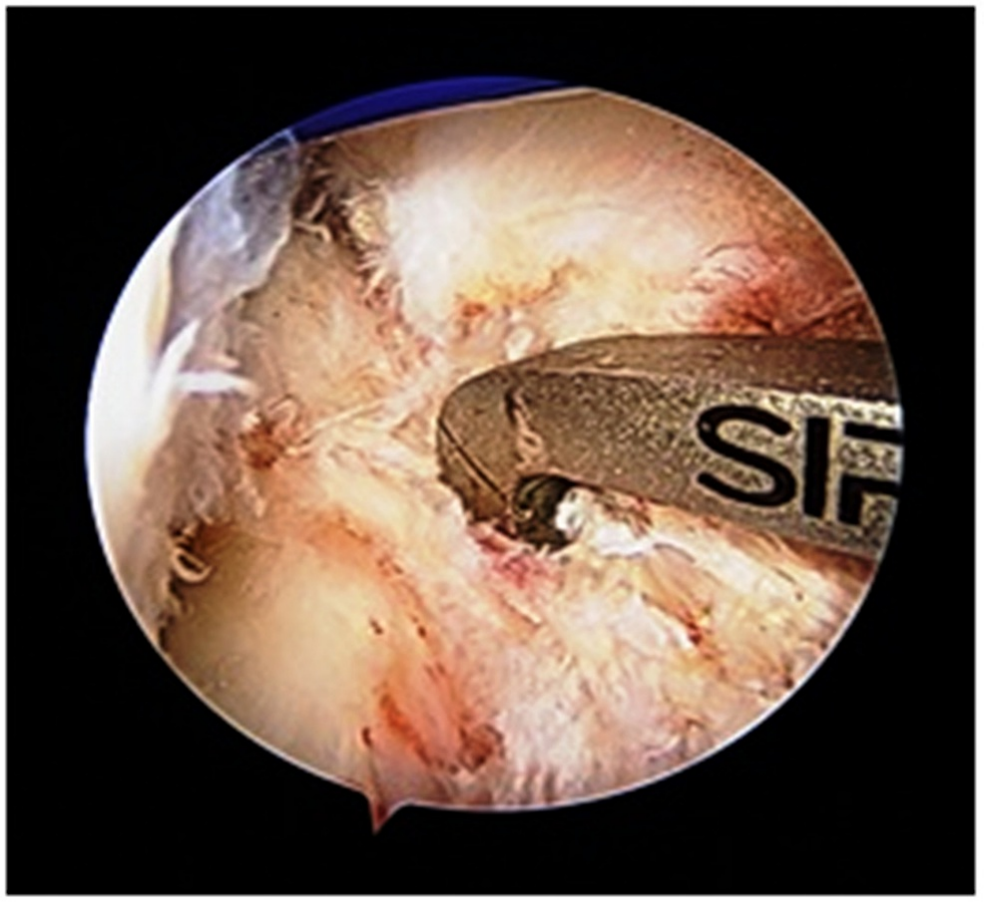
Exit point of the tibial canal

Semitendinosus and gracilis muscle tendons were delivered out by a 4-cm longitudinal incision positioned over the anteromedial aspect of the tibia from tibial tuberosity directing downward. The quadrupled graft was prepared after denuding all muscle fibers and stitching the end with Ethibond sutures (Figure 3). The diameter and length of the prepared graft were calculated. For better visibility of the intercondylar notch in full knee flexion, the excessive fat pad was excised. The tibial footprint of ACL was excised using a shaver. ACL attachment over the femoral footprint was preserved. Using an IV cannula needle or a spinal needle, an AAM was made by clearing the lateral margin of the medial femoral condyle and just above the medial meniscus. The femoral tunnel guide pin was placed in the center of the femoral footprint. The guide pin position was checked through the medial portal. The femoral tunnel was made by drilling with a properly sized reamer equal to graft diameter through AAM. Reaming was done gently and with a properly sized reamer so that the lateral femoral cortex was not compromised. The tibial canal for graft was drilled using a standard ACL tibial jig at an angle of 55° from the tibia such that the exit point was posterior and just lateral to the posterior border of the anterior horn of the lateral meniscus (Figure 4).

**Figure 5:**
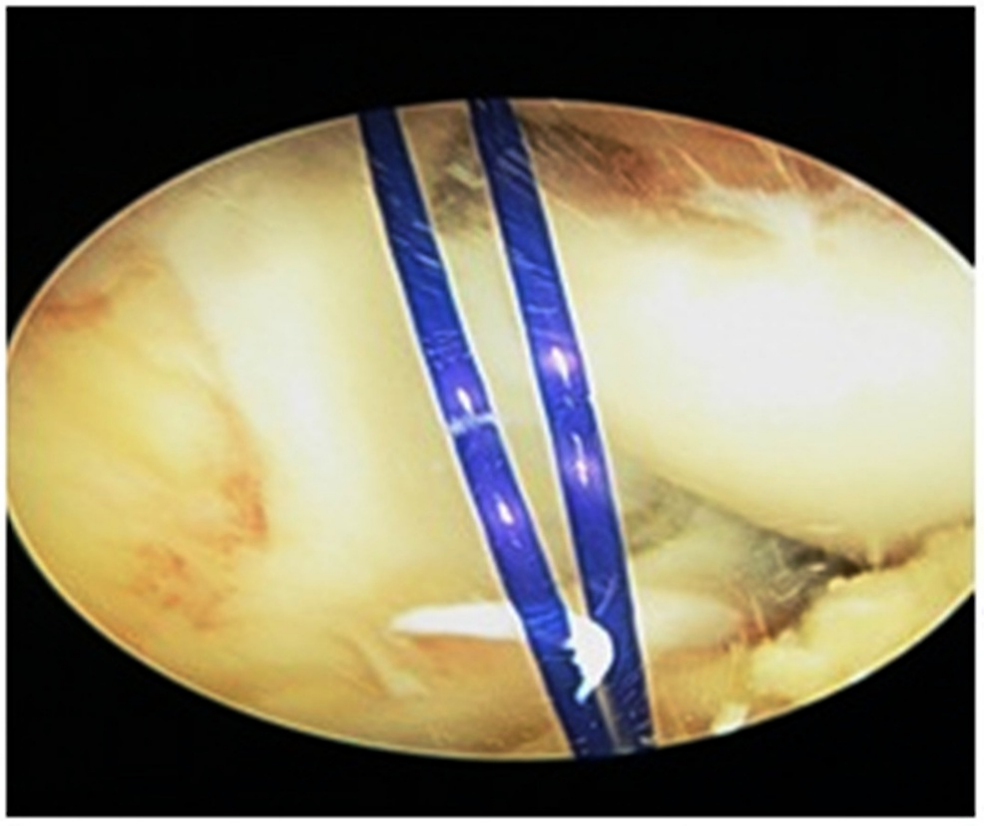
Proline sutures used for graft passage

**Figure 6:**
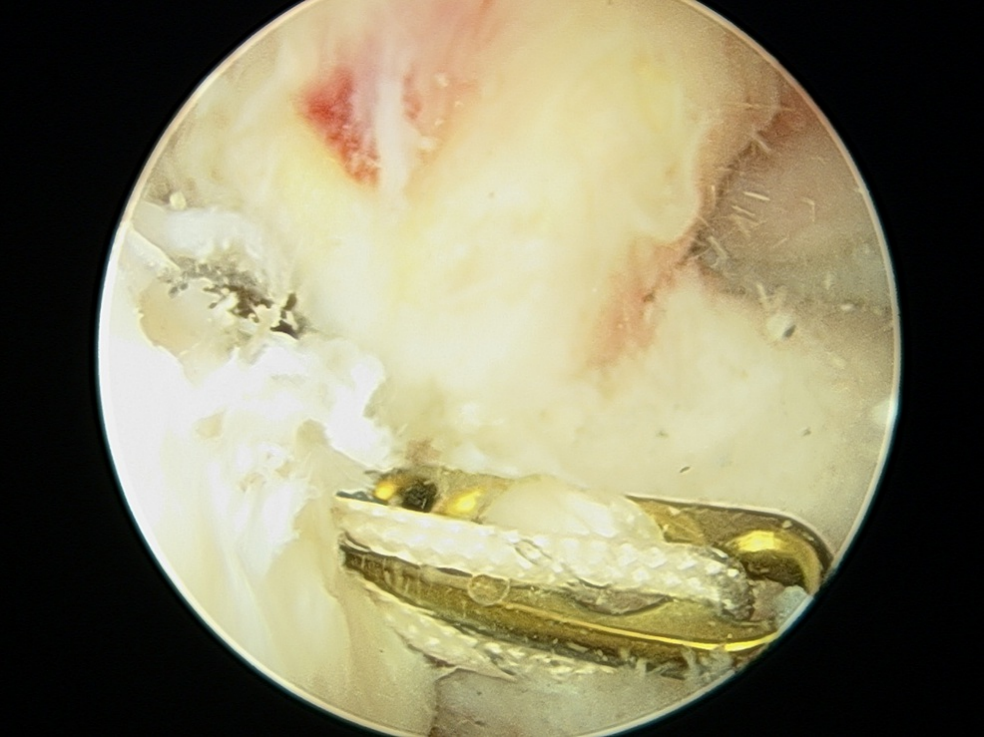
Graft passage with Endobutton

**Figure 7:**
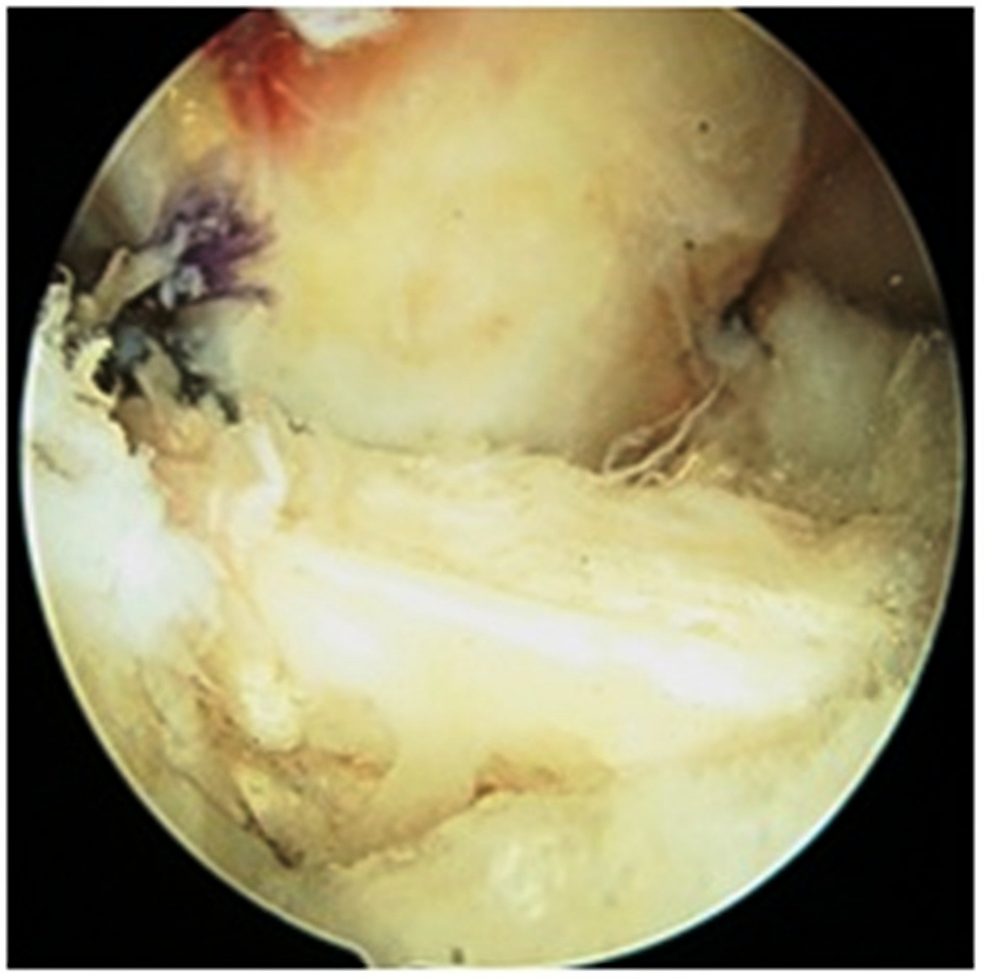
Final graft placement

**Figure 8:**
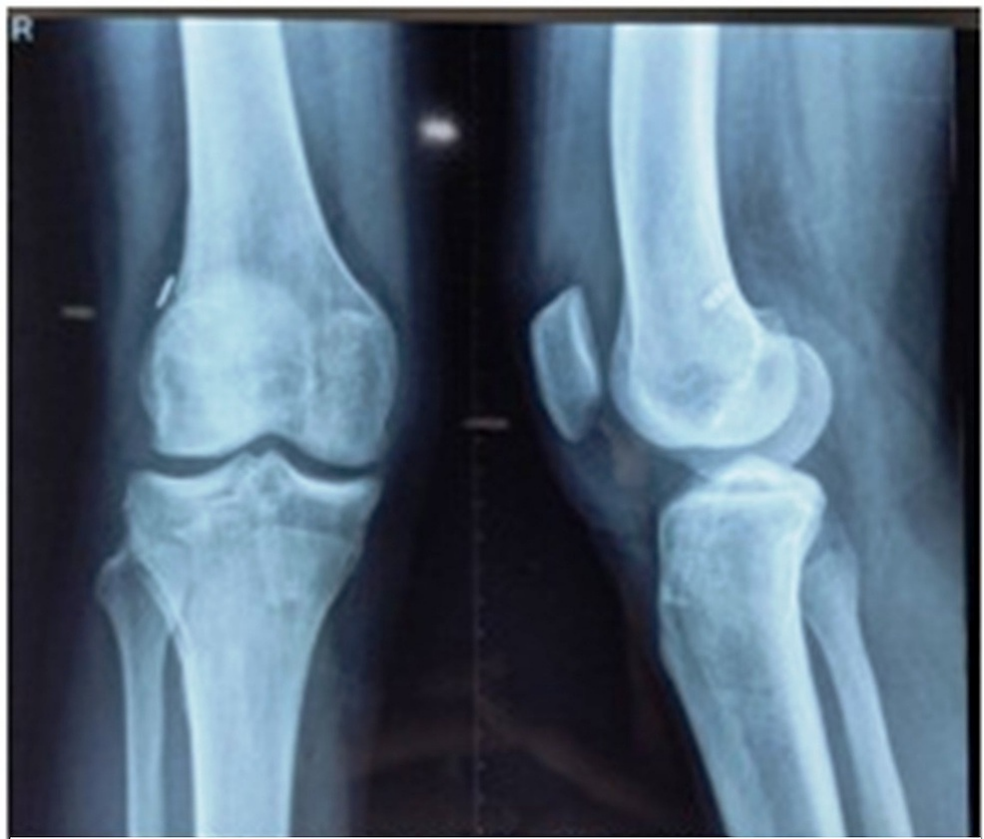
Postoperative radiograph with Endobutton

For graft passage, proline sutures were used (Figure 5). The graft was negotiated from the tibial tunnel to the femoral tunnel with the aid of moving sutures (Figure 6). The graft was fixed on the femoral side with an Endobutton and on the tibial side with an acceptably sized bioabsorbable interference screw. After visualizing the final placement of the graft, a cycling maneuver was performed 20-25 times (Figure 7). Postoperative X-ray showed the proper placement of femoral and tibial tunnel and Endobutton (Figure 8). All the patients were advised to use a functional knee range of motion (ROM) brace. Static quadriceps and hamstring exercises were started on the very next day after the operation. Weight-bearing was introduced in patients after adequate quadriceps strength was achieved. Tegner-Lysholm scoring was performed at one month, three months, six months, and one-year follow-ups. Full knee extension was achieved in all patients after a month. Patients were allowed to return to sports activities after achieving good quadriceps and hamstring muscle power.

## Results

**Figure 9:**
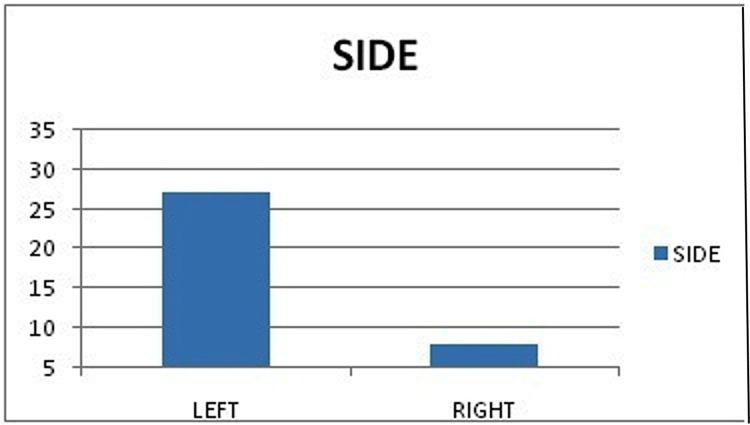
Graph showing that left side involvement was more predominant in the study group

A total of 38 patients with ACL tears were operated on. Among them, three patients were lost to follow-up and were excluded from the study. Patients were followed up at regular intervals for a period of one year. The mean age of the patients was 27.76 years (range: 18-45 years). The male-to-female ratio was 2.5:1 (Table 1). ACL injury in the left side was found to be present in 27 (77.14%) patients in our study (Figure 9); 54.28% of patients had a history of road traffic accidents (RTAs), which had caused the ACL tears (Table 2).

**Table 1:** Demographic profile of patients in the study

Age group (years)	Males (n=25)	Females (n=10)	Total (n=35)
18-20	2	1	3
21-30	14	4	18
31-40	5	3	8
41-45	4	2	6

**Table 2:** The mode of injuries in the study group

Mode of injury	Number	Percentage
Road traffic accident	19	54.28%
Sports	15	42.85%
Fall from height	1	2.87%
Total	35	100%

**Figure 10:**
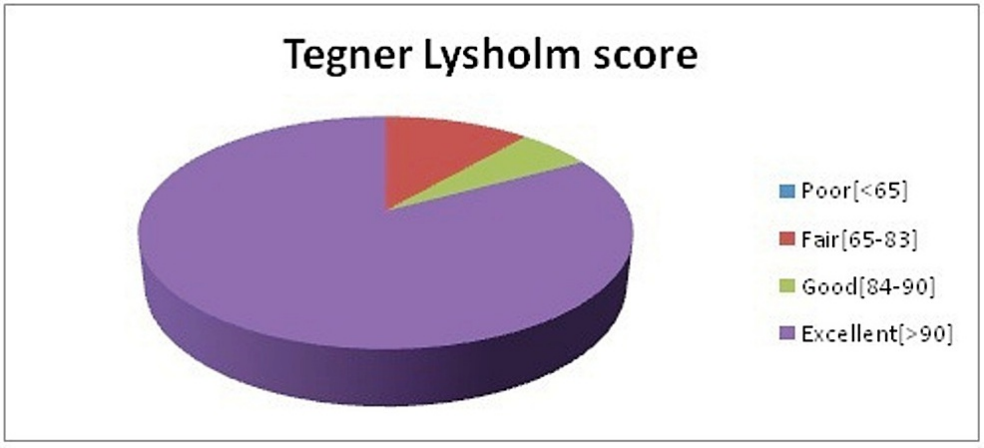
Postoperative (at three-month follow-up) Tegner-Lysholm scores in the study group

In our study, we found that the chief complaint of patients was instability (80%), followed by pain (14.28%), and locking sensation (5.72%) (Table 3). Lateral meniscus injury requiring repair was found in 68.57% of our patients (Table 4). But the average duration post-trauma till the presentation of patients was approximately eight weeks, and hence meniscus repair surgeries were not opted for, and appropriate meniscus balancing was done in such cases. Tegner-Lysholm scores were compared before and after the operations. In our study, 19 (54.28%) patients had poor, and 16 (45.72%) patients had fair Tegner-Lysholm scores before the operation. Postoperatively, at the three-month follow-up, 29 (82.85%) patients had excellent, and two (5.71%) had a good score, and the other four (11.44%) had a fair score as per Tegner-Lysholm scoring system (Figure 10). Overall, three patients had complications; knee pain and thigh muscle wasting [2-3 cm] were present in all three, and a sense of giving away during exertion was present in two out of three (Table 5), which were not further investigated and managed by physiotherapy and analgesics. At the one-year follow-up, 33 patients had excellent and two patients had good Tegner-Lysholm scores. We observed that the usage of AAM provided ease to surgeons for anatomical graft placement at the femoral footprint, resulting in shorter operative times and good functional outcomes.

**Table 3:** Frequency of presenting complaints in the study group

Presenting complaint	Frequency (n)	Percentage
Instability	28	80%
Pain	5	14.28%
Locking	2	5.72%

**Table 4:** Frequency of meniscus injury along with ACL tear in the study group ACL: anterior cruciate ligament

Associated meniscus injury	Frequency (n)	Percentage
Lateral meniscus	24	68.57%
Medial meniscus	8	22.85%
Both meniscus	3	8.58%

**Table 5:** Complications encountered in the study group

Complications	Number of patients
Infection	0
Effusion of joint	0
Knee pain	3
Sense of giving away during exertion	2
Graft failure	0
Graft site tenderness	0
Muscle wasting	3

## Discussion

The primary goals of arthroscopic ACL reconstruction are to give good knee ROM, enabling patients to get back to their pre-injury levels, and avoiding further injury to the knee. Freedman et al. [10], Goldblatt et al. [11], Charlton et al. [12], and Anderson et al. [13] in their studies have reported good functional outcomes after ACL reconstruction by quadrupled hamstring graft. The hamstring graft is commonly used for anatomical ACL reconstruction currently. Many studies have shown good results with the least complications and excellent patient satisfaction both in terms of physical activity and ratings on a pain scale [10,11,12,13].

The sole aim of this study was to assess the anatomical arthroscopic ACL reconstruction, which is essential for good knee kinematics and hence better functional outcomes. In most of the literature, the emphasis is placed more on anatomic tunnel positioning [14]. Therefore, ACL reconstruction was recommended via an AAM portal built just above the medial meniscus for drilling the femoral tunnel because it eases the visualization of the femoral footprint, thereby enabling better identification of the anatomical position for tunnel placement. We used the AAM portal to drill the femoral tunnel in our research as well.

The main outcome measure of this study was patients with stable painless knees with fewer complications and a faster return to their pre-injury levels. In our study, the average age of individuals with ACL injury was 27.76 years, which was comparable with other studies. In a study by Specchiulli et al. [15], it was 27 years, and it was 26.8 years and 26 years in studies by Chaudhary et al. [16] and Jomha et al. [17], respectively.

In our study, males were injured more as compared to females. This can be attributed to the fact that females are less involved in sports activities in rural settings and also the underreporting of injuries among females.

Our study showed that RTAs were the predominant cause of injuries, followed by sports-related injuries. Patond et al. [18] found that sporting practices were the primary cause of ACL trauma. Sports activities were responsible for 66.7% of injuries while RTA and household activities were responsible for 30.8% and 2.5%, respectively in the study conducted by Chaudhary et al. [16]. Most studies have reported sports-related injury as the main cause of an ACL tear. But our study revealed a higher rate of injuries caused by RTAs due to the careless attitude toward driving by a mostly young population and related injuries.

In our study Tegner-Lysholm knee scoring at the three-month follow-up was excellent in 29 (82.85%) patients. In their study, Sun et al. [19] reported a median Lysholm score of 92 at two-year follow-up.

Complication rates in our study were minimal, with knee pain, sense of giving away, and thigh muscle wasting found only in a few patients, which were not so significant. A follow-up MRI can be done for proper evaluation of such complications, which was lacking in our study. Deehan et al. [20] have also reported muscle wasting of less than 1 cm in 87% of patients, 1-2 cm in 9%, and 3 cm in 4% in their study.

## Conclusions

Albeit the study conducted was not a randomized analysis, and the sample size was small, we found that the anatomical reattachment of tendon graft for ACL reconstruction at femoral and tibial footprints is indispensable for good functional outcome and knee kinematics; our findings also showed that the usage of AAM provides good visualization of femoral footprint and ease to surgeons during ACL reconstruction for making near-accurate femoral tunnels, thereby helping to achieve better outcomes.
